# Constitutive Interleukin-7 Cytokine Signaling Enhances the Persistence of Epstein–Barr Virus-Specific T-Cells

**DOI:** 10.3390/ijms242115806

**Published:** 2023-10-31

**Authors:** Sandhya Sharma, Tim Sauer, Bilal A. Omer, Thomas Shum, Lisa A. Rollins, Cliona M. Rooney

**Affiliations:** 1 Graduate Program in Translational Biology and Molecular Medicine, Baylor College of Medicine, Houston, TX 77030, USA; sandhyas@bcm.edu (S.S.);; 2 Center for Cell and Gene Therapy, Texas Children’s Hospital, Baylor College of Medicine, Houston, TX 77030, USA; 3 Department of Pediatrics, Section of Hematology-Oncology, Baylor College of Medicine, Houston, TX 77030, USA; 4 Department of Molecular Virology and Microbiology, Baylor College of Medicine, Houston, TX 77030, USA; 5 Department of Pathology-Immunology, Baylor College of Medicine, Houston, TX 77030, USA

**Keywords:** EBV-specific T-cells (EBVSTs), immunotherapy, virus-specific T-cells (VSTs)

## Abstract

The efficacy of therapeutic T-cells is limited by a lack of positive signals and excess inhibitory signaling in tumor microenvironments. We previously showed that a constitutively active IL7 receptor (C7R) enhanced the persistence, expansion, and anti-tumor activity of T-cells expressing chimeric antigen receptors (CARs), and C7R-modified GD2.CAR T-cells are currently undergoing clinical trials. To determine if the C7R could also enhance the activity of T-cells recognizing tumors via their native T-cell receptors (TCRs), we evaluated its effects in Epstein–Barr virus (EBV)-specific T-cells (EBVSTs) that have produced clinical benefits in patients with EBV-associated malignancies. EBVSTs were generated by stimulation of peripheral blood T-cells with overlapping peptide libraries spanning the EBV lymphoma antigens, LMP1, LMP2, and EBNA 1, followed by retroviral vector transduction to express the C7R. The C7R increased STAT5 signaling in EBVSTs and enhanced their expansion over 30 days of culture in the presence or absence of exogenous cytokines. C7R-EBVSTs maintained EBV antigen specificity but were dependent on TCR stimulation for continued expansion. C7R-EBVSTs produced more rapid lymphoma control in a murine xenograft model than unmodified EBVSTs and persisted for longer. The findings have led to a clinical trial, evaluating C7R-EBVSTs for the treatment of refractory or relapsed EBV-positive lymphoma (NCT04664179).

## 1. Introduction

Epstein–Barr Virus (EBV) is linked to a range of malignancies including about 30% of Hodgkin and non-Hodgkin lymphomas in which four viral latent cycle proteins, EBNA-1, LMP1, LMP2, and BARF1 (Type 2 latency antigens (T2-Ags)) are expressed [1,2,3]. Although EBV-specific T-cells (EBVSTs) are effective against post-transplant lymphoproliferative disease (PTLD) [4,5], their efficacy against the less immunogenic and more immunosuppressive T2 lymphomas is limited by difficulties in reactivating and expanding EBVSTs from patients [6], reduced viral target antigen expression [7], and a lack of T-cell expansion and persistence following infusion [8,9]. In our clinical trial using EBVSTs to treat refractory/relapsed lymphoma (NCT01555892), we observed only transient increases in the frequency of EBVSTs in the blood of most patients as measured in enzyme-linked immunospot (ELIspot) assays that measure the number of cells that secrete γIFN in response to T2 antigen stimulation. Increases in the frequency of EBVSTs were accompanied in responding patients by epitope spreading, defined as an increase in the frequency of T-cells specifically for the non-viral tumor antigens MAGEA4, NY-ESO-1, survivin, and SSX2, a phenomenon that is likely important for the clinical success of EBVSTs [9]. The goal of our research was to increase the expansion and persistence of EBVSTs in patients by providing intrinsic cytokine stimulation.

The expansion and survival of adoptively transferred T-cells is limited by the suppressive tumor-microenvironment (TME) [10,11,12] that contains immunosuppressive cytokines and chemokines that recruit or induce tumor-associated macrophages, cancer-associated fibroblasts, regulatory T-cells, and other immunosuppressive cells, while a lack of proinflammatory cytokines such as interleukin (IL) Il-2, IL-7 and IL-15 [11,13] starves tumor-specific T-cells of the cytokines needed for proliferation and survival. While this harsh environment is considered a hallmark of solid tumors, hematological malignancies such as lymphoma are similarly immunosuppressive, producing IL-10, TGFβ, and IL-13, among other inhibitory ligands that promote tumor growth and inhibit effector T-cells [14,15,16].

The early success of high-dose IL-2 cytokine administration along with tumor-infiltrating lymphocytes (TILs) [17,18] established the essential role of immunostimulatory cytokines to promote the expansion and persistence of adoptively transferred T-cells. While various preclinical studies have evaluated a plethora of cytokines: Il-7, IL-15, IL-12, IL-18, and their role in enhancing the functional survival and persistence of T-cells in the context of adoptive immunotherapy and clinical translation of systemic administration of cytokines is hindered by their lack of restriction to tumor milieu, and adverse toxic effects [19,20]. To avoid the toxicity of exogenous cytokines, lymphodepleting chemotherapy prior to therapeutic T-cell infusion is commonly used to provide T-cell growth-promoting cytokines, since in the absence of endogenous lymphocytes, the homeostatic cytokines, IL-7 and IL-15 are available for adoptively transferred T-cells [21,22]. Although lymphodepletion enables dramatic expansion of tumor-infiltrating lymphocytes (TILs) [23] and a chimeric antigen receptor (CAR) expressing T-cells [24,25,26], the effects are transient and likely also destroy endogenous TILs and other tumor-reactive T-cells that are the source of epitope spreading. To restrict cytokines to the tumor vicinity, several studies have expressed IL-15, IL-12, or IL-18 as transgenes in CAR-T or NK-cells [27,28,29], or have loaded T-cells with IL-15 containing nano gel “backpacks” that are released following TCR activation [30]. However, secreted cytokines are restricted not only to effector T-cells, but might also promote inhibitory cells or even tumor cells in the TME.

To enhance the proliferation, survival, and persistence of adoptively transferred EBVSTs, we have focused on providing IL-7 cytokine signaling. To circumvent the toxicity and limitations associated with secreted cytokines, we used a constitutively active IL-7 receptor (IL7R). Cysteine and/or proline insertions in the transmembrane domain of the IL7R α chain produces homodimerization resulting in phosphorylation of JAK1 and activating STAT5 signaling [31,32]. To evaluate this concept, our lab designed a constitutively active IL7R (C7R) and demonstrated enhanced persistence and anti-tumor activity of C7R-modified CAR T-cells in xenograft models of neuroblastoma and glioblastoma [33]. This strategy was validated by Zhao et al. who demonstrated enhanced persistence and anti-tumor efficacy of C7R-expressing CAR T-cells in preclinical triple-negative breast cancer-cell models [34]. We have now evaluated the C7R construct in EBVSTs targeting EBV+ lymphoma via their native TCRs. C7R enhances EBVST proliferation and maintains EBV antigen specificity in vitro and produces superior in vivo anti-tumor efficacy in our murine EBV+ xenograft model, correlating with enhanced persistence compared to unmodified EBVSTs.

## 2. Results

### 2.1. Constitutive STAT5 Activation in EBVSTs Expressing the Constitutively Active IL7 Receptor (C7R)

**Figure 1:**
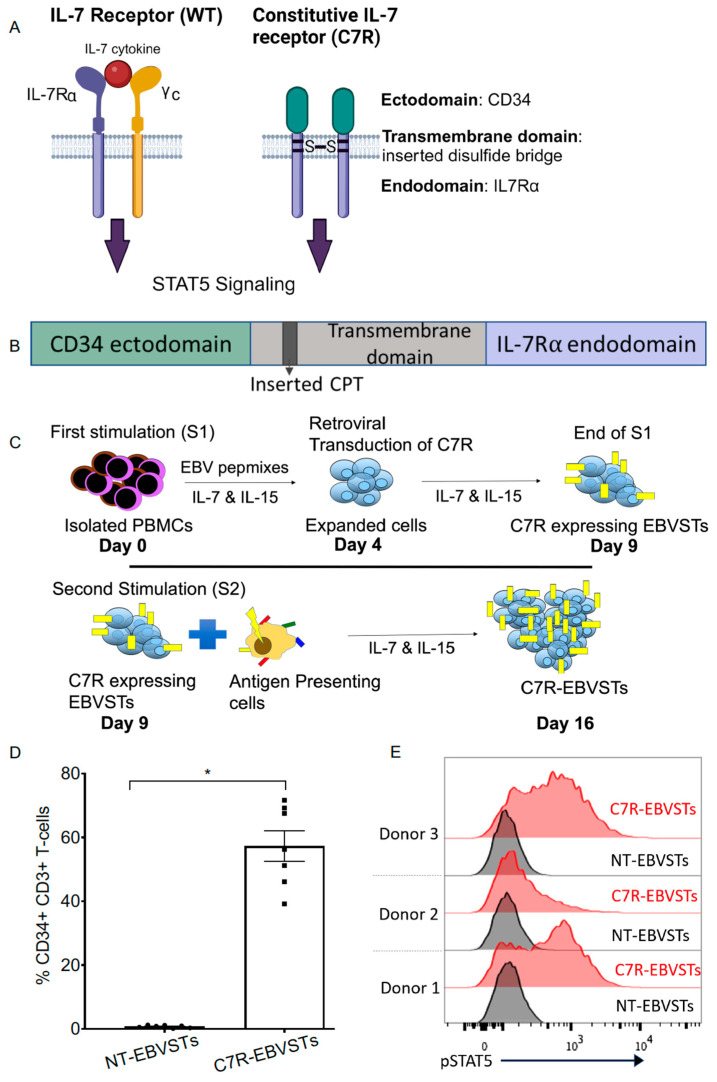
Retroviral transduction of C7R expressing EBVSTs. (**A**) Representation of wild-type (WT) IL-7 receptor showing IL-7 cytokine binding to IL-7 receptor (IL7Rα and common γc heterodimer) compared to the engineered constitutively active IL-7 receptor (C7R) homodimerized receptor. (**B**) The C7R constitutes a CD34 ectodomain to prevent the constitutive receptor acting as a sink for IL-7 and to allow the detection of transduced T cells using human CD34 antibody, cysteine, proline, and threonine insertions between Thr244 and Ile245 in the IL7Rα transmembrane domain to create a disulfide bridge and the IL7Rα endodomain (created with BioRender.com) [33] CPT = cysteine, proline, threonine. (**C**) Schematic showing retroviral-mediated transduction of EBVSTs to generate C7R-modified EBVSTs (C7R-EBVSTs). EBVSTs were generated as described in the materials and methods section and retrovirally transduced with the C7R retrovirus supernatant on day 4 of culture followed by culture for five more days. On day 9, EBVSTs received a second stimulation with an irradiated antigen-presenting complex comprising autologous ATCs pulsed with pepmixes and ULCL in the presence of IL-7 and IL-15 cytokines to expand EBVSTs. Unless otherwise indicated, cell characterization was performed on day 16. (**D**) C7R-EBVSTs were stained with CD34 antibody to evaluate C7R transduction efficiency and analyzed by flow-cytometry (n = 7). (**E**) Mean fluorescence intensity measured for STAT5 phosphorylation in C7R-EBVSTs and non-transduced (NT) EBVSTs. On day 16, T-cells were harvested, washed, and cultured without IL-7 and IL-15 for 72 h before STAT5 analysis (n = 3). Statistical comparisons were determined using paired two-tailed Student’s *t*-test. *p* < 0.05 (*). Data shown are plotted as mean ± SEM.

The C7R comprises a CD34-derived ectodomain, linked to the transmembrane and intracellular domains of the IL7Rα chain, carrying in the transmembrane domain that results in homodimerization as outlined in Figure 1A,B [33]. EBVSTs were generated from EBV seropositive donor peripheral blood mononuclear cells (PBMCs) by stimulation with overlapping EBV peptide libraries (15 amino acids overlapping by 11 amino acids) spanning the entire protein sequences of type-2 EBV latent antigens EBNA-1, LMP-1, and LMP-2 in the presence of IL-7 and IL-15. On day 4, the cells were transduced with a retroviral vector expressing the C7R and cultured in the continued presence of cytokines. On day 9 after the first stimulation (S1), the cells were re-stimulated (S2) using an irradiated antigen-presenting complex comprising pepmix-pulsed, autologous activated T-cells (ATCs) plus an HLA negative lymphoblastoid cell line (ULCL) that provides costimulatory signaling to T-cells [6] in the presence of IL-7 and IL-15 cytokines. On day 16, non-transduced (NT)- and C7R-EBVSTs were harvested and used in functional studies (Figure 1C).

### 2.2. C7R-EBVSTs Maintain EBV Antigen Specificity and Cytotoxic Function

**Figure 2:**
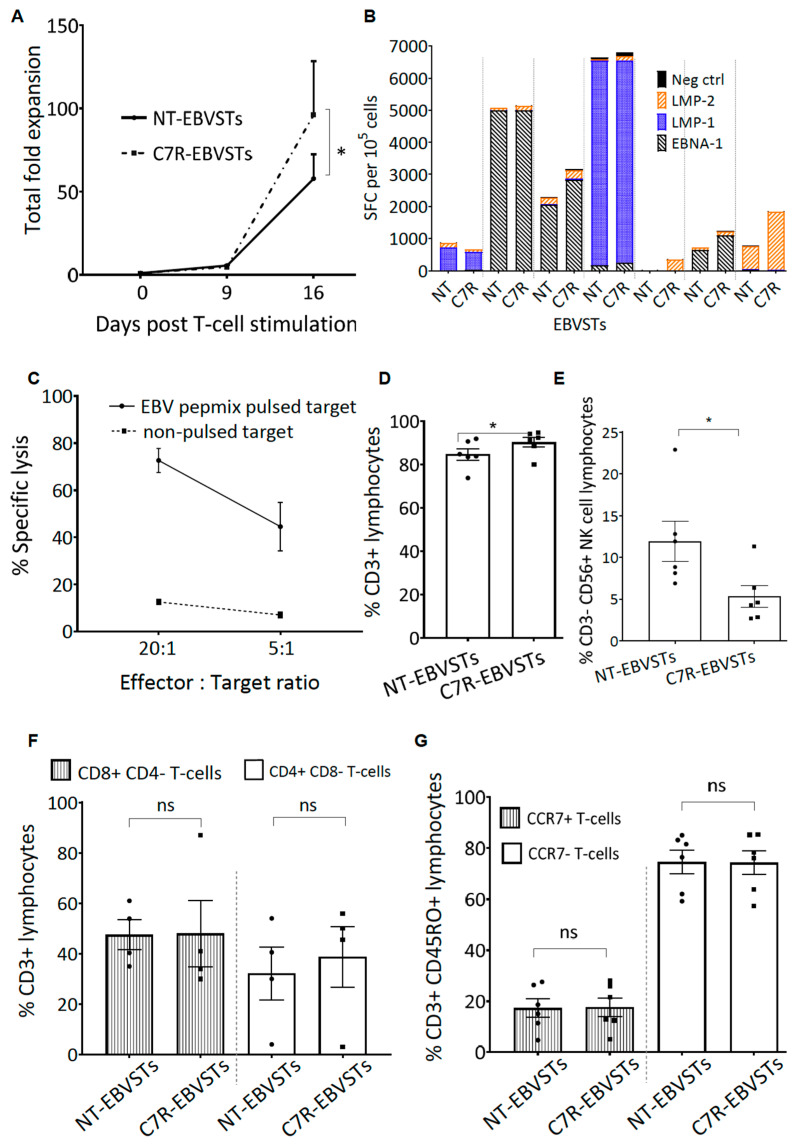
Functional characterization of C7R expressing EBVSTs. C7R-EBVSTs and NT-EBVSTs were generated from 7 EBV seropositive healthy donors. (**A**) Total fold expansion from day 0 to day 16. (n = 6). (**B**) Antigen-specificity on day 16. *Y*-axis shows the number of cells per 10^5^ EBVSTs that produced IFNγ in response to stimulation with EBV antigens in an ELISpot assay. SFC = spot-forming cells. The dotted line separates data from each donor (n = 7). (**C**) Killing of EBV pepmix-pulsed and unpulsed autologous activated T-cells by C7R-EBVSTs in a 4 h chromium-release assay at an effector: target cells ratio of 20:1 and 10:1 (n = 6). Frequency of (**D**) CD3+ T-cells (**E**) CD3− CD56+ NK-cells (**F**) % of CD3+ CD8+ CD4− vs. CD3+ CD8− CD4+ and (**G**) CD3+ CD45RO+ CCR7+ central memory (T_CM_) vs. CD3+ CD45RO+ CCR7- effector memory (T_EM_) T-cells in C7R- and NT-EBVSTs (n = 6). Statistical comparisons were determined using paired two-tailed Student’s *t*-test on day 16 fold expansion *p* < 0.05 (*), ns: non-significant. The data shown are plotted as mean ± SEM.

To assess the functional potency of C7R-EBVSTs, we evaluated their proliferation, antigen specificity, and phenotype on day 16 of culture (Figure 2). C7R-EBVSTs proliferated to a greater extent in the presence of cytokines than NT-EBVSTs (total fold expansion; 87.5 ± 28.78 vs. 51.32 ± 13.96 [mean ± SEM, *p* = 0.047]) over 16 days (Figure 2A). C7R-EBVSTs maintained their specificity to the EBV T2-Ags as evaluated in IFNγ ELIspot assays that measure the number of cells per 10^5^ cells that produce IFNγ in response to antigen stimulation (Figure 2B) (of note, antigen specificity as measured in ELIspot assays underestimates the frequency of antigen-specific T-cells by at least 1 log) [35]. The antigen specificity of C7R-EBVSTs was similar to that of NT-EBVSTs in 2/7 donors, diminished in one, and increased in four (Figure 2B). C7R-EBVSTs also demonstrated antigen-specific killing of EBV, pepmix-pulsed, autologous target cells in ^51^Cr chromium release assays (Figure 2C) while showing minimal to no cytotoxicity against unmodified autologous target cells. We observed a higher frequency of CD3+ T-cells in C7R-EBVSTs (90.23% ± 2.21% vs. 84.75% ± 2.64%, mean ± SEM, C7R- vs. NT-EBVSTs, *p* = 0.031) (Figure 2D), which correlated with a decreased frequency of CD3-CD56+ NK-cells (5.32% ± 1.4% vs. 11.9% ± 2.4%, mean ± SEM, C7R- vs. NT-EBVSTs, *p* = 0.031) (Figure 2E) indicating preferential expansion of C7R-expressing CD3+ T-cells. There was no significant difference in the CD4+ to CD8+ ratio (Figure 2F), or in the frequency of CD45RO+, CCR7+ central memory (T_CM_), or effector memory (T_EM_) (CD45RO+ CCR7-) CD3+ T-cells (Figure 2G), allaying fears that increased proliferation would result in increased T-cell differentiation. These observations establish the successful generation of C7R-expressing EBVSTs and their target antigen-specific cytotoxicity.

### 2.3. C7R Enhances the Survival and Specificity of EBVSTs in the Absence of Cytokines

**Figure 3:**
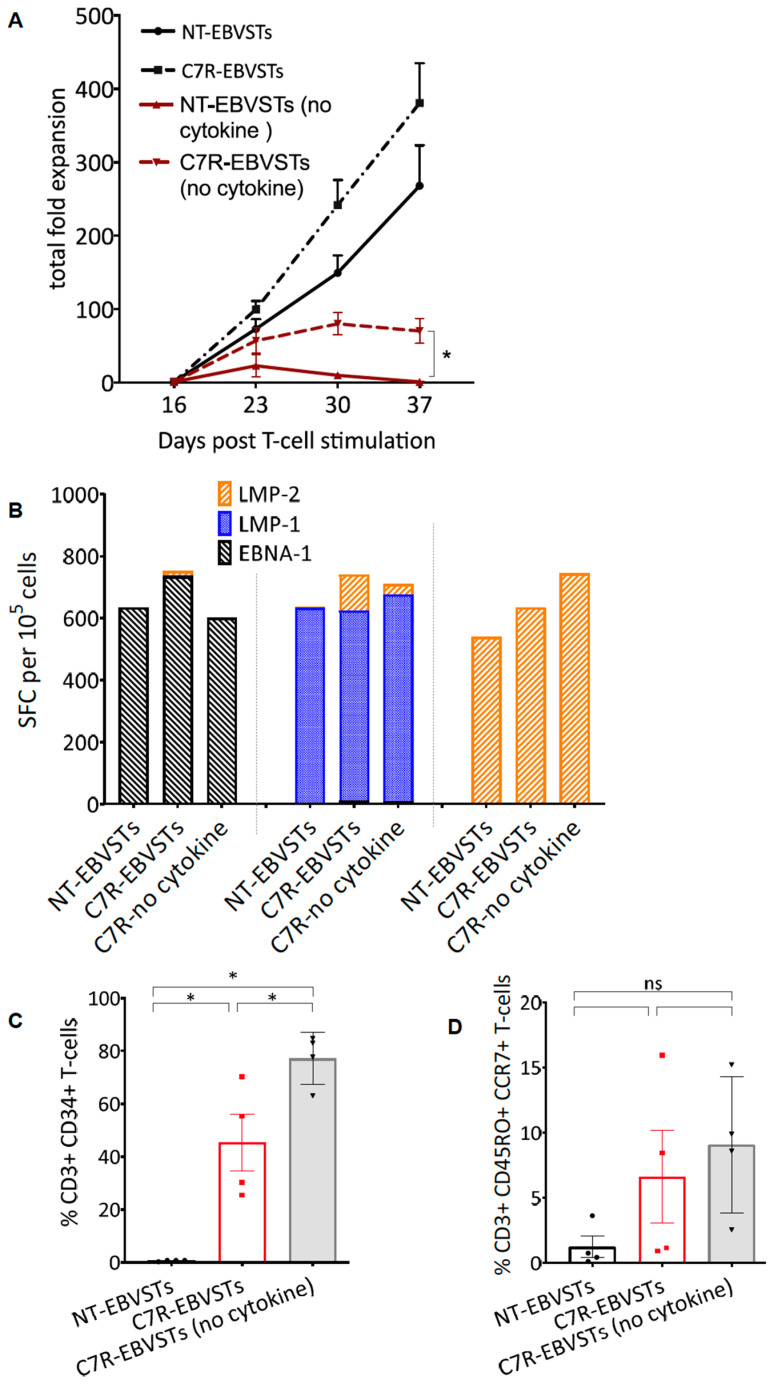
Long-term culture of C7R-EBVSTs in the absence of exogenous cytokines administration. From day 16, C7R- and NT-EBVSTs were restimulated weekly with the irradiated antigen-presenting cells complex used for S2 with or without IL-7 and IL-15 that were replenished every 2 to 3 days. (**A**) Total fold expansion of EBVSTs from day 16 to 37. Day 16 was considered the starting time point for this analysis (n = 3) Statistical comparisons were determined using paired two-tailed Student’s *t*-test on day 16, fold expansion *p* < 0.05, (0.12 (ns: non-significant), 0.033 (*). The data shown are plotted as mean ± SEM. (**B**) Antigen-specificity on day 30 as measured by ELIspot assay. *Y*-axis shows the number of cells per 10^5^ EBVSTs that produced IFNγ in response to stimulation with EBV antigens (n = 3). (**C**,**D**) Flow cytometric analysis was conducted on day 30 to evaluate the % frequency of (**C**) CD3+ CD34+ T-cells to determine the frequency of C7R expressing EBVSTs and (**D**) CD3+ CD45RO+ CCR7+ T-cells to evaluate their surface CCR7 expression.

To determine if C7R can replace exogenous cytokines, we evaluated its effects on the long-term proliferative capacity and antigen-specificity of EBVSTs in the absence of exogenous cytokines. From day 16, we restimulated EBVSTs weekly, with the irradiated antigen-presentation complex used for the second stimulation in the presence or absence of IL-7 and IL-15. While both NT- and C7R-EBVSTs continued to proliferate in response to weekly antigen stimulation in the presence of IL-7 and IL-15 (Figure 3A), NT-EBVSTs failed to proliferate when stimulated in the absence of exogenous cytokine administration, and cell numbers decreased from day 23 to day 30. In contrast, while the proliferation of C7R-EBVSTs was diminished without cytokine supplementation, they continued to expand until day 30 before decreasing in number and viability (Figure 3A). This diminished proliferation could result from a lack of IL-15 production; despite the supplementation of Il-7 cytokine signaling via C7R. C7R-EBVSTs maintained their EBV antigen specificity and C7R expression (Figure 3C) in the absence of cytokines as measured in the IFN-γ ELIspot assays (Figure 3B). Indeed, as expected, the frequency of CD3+ CD34+ C7R-expressing T-cells was significantly greater in C7R-EBVSTs cultured without exogenous cytokines than in C7R-EBVSTs cultured with cytokines (*p* = 0.044, Figure 3C). We also observed a trend towards a higher frequency of CCR7+ CD45RO+ expressing T_CM_-cells in C7R-EBVSTs cultured both in the presence or absence of cytokines compared to NT-EBVSTs, although this was not statistically significant (Figure 3D). In summary, in the absence of cytokines, C7R expression prolongs the in vitro survival of EBVSTs while maintaining their antigen specificity and effector function.

### 2.4. C7R Increases the Persistence and Anti-Tumor Activity of EBVSTs In-Vivo

**Figure 4:**
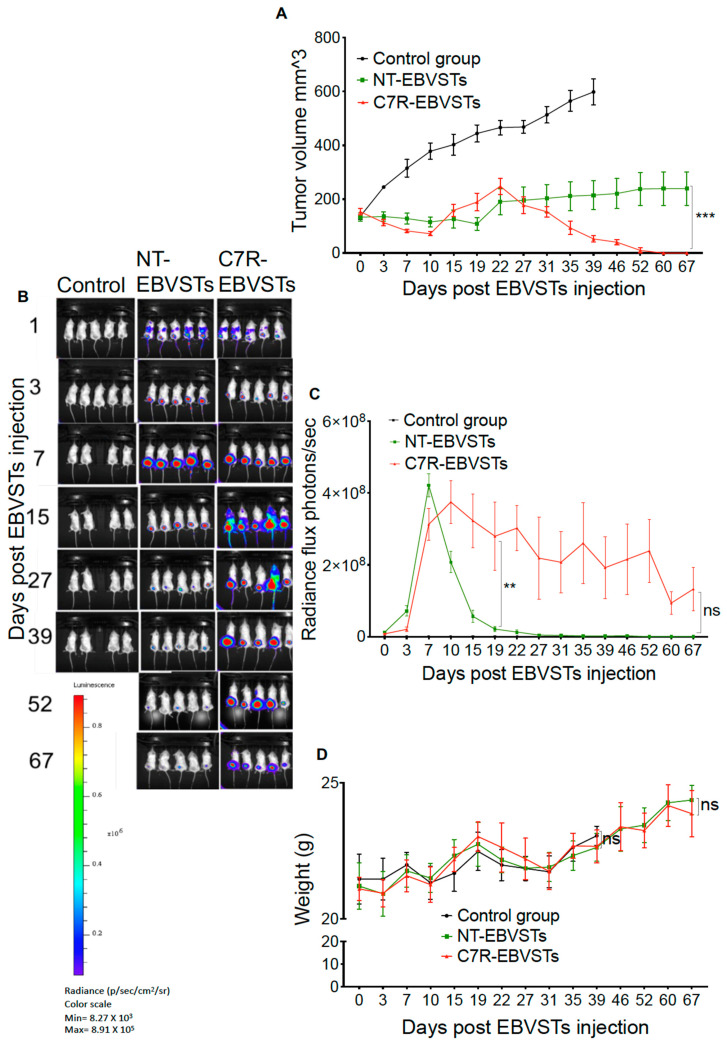
In-vivo functional characterization of C7R- and NT-EBVSTs. EBV-LCLs (2.5 × 10^6^) were suspended in 200 μL Matrigel and injected subcutaneously into the left flanks of NSG mice. Ten days later, when tumors were palpable, 2.5 × 10^6^ GFP-ff-luc expressing autologous NT- or C7R-EBVSTs were infused via tail vein injection. Control mice were injected with 200 μL of sterile PBS only (n = 5 mice per group). (**A**) Tumor volume as measured using calipers, and calculated using the equation: tumor volume = ½ (length) × (width)^2^. (**B**) Quantitative radiance flux of T-cell bioluminescence of the tumor area (photons/sec) per time point. (**C**) Bioluminescence imaging at the time points indicated after EBVST injection. (**D**) Mice weight in grams. Data shown are plotted as mean ± SEM. Statistical analysis was performed for particular time points using two-way ANOVA analysis with Bonferroni correction *p* < 0.01 (**), *p* < 0.001 (***), ns: non-significant, to compare the bioluminescence and tumor volume in mice receiving NT and C7R-EBVSTs.

To evaluate the persistence and anti-tumor effects of C7R-modified EBVSTs in vivo, we implanted EBV-transformed B lymphoblastoid cell lines (LCLs) subcutaneously in NSG mice. Ten days after implantation, when tumors were palpable, we adoptively transferred 2.5 × 10^6^ autologous GFP-Firefly-luciferase (GFP-ff-luc) expressing NT- or C7R-EBVSTs, and measured tumor volume and T-cell bioluminescence to evaluate anti-tumor efficacy and T-cells’ persistence over time. Tumor clearance, as determined by a lack of palpable tumor, was observed on day 67 after C7R-EBVST infusion. At this time, tumors measured 239.49 mm^3^ ± 61.96 mm^3^ in mice receiving NT-EBVSTs (Figure 4A). The superior anti-tumor efficacy of C7R-EBVSTs correlated with enhanced T-cell persistence after day 7 as indicated by bioluminescence imaging (Figure 4B,C). The increased radiance flux of C7R-EBSVTs on day 22 post EBVSTs infusion compared to that of NT-EBVSTs (*p* = 0.004) (Figure 4C) corresponds to a time-point after which tumor volume began to decrease. C7R-EBVST radiance eventually declined to insignificance by day 67 (Figure 4B,C) indicating a lack of in-vivo autonomous growth. Weight loss or other signs of toxicity were not observed in any treatment group (Figure 4D).

## 3. Discussion

The lymphoma TME is not only immunosuppressive but lacks immunostimulatory cytokines critical to EBVST survival and persistence [13]. The constitutively active IL-7 receptor (C7R) provided a cytokine signal that enhanced the persistence and function of adoptively transferred EBVSTs. C7R-EBVSTs demonstrated increased levels of phosphorylated STAT5, a downstream molecule in the IL-7 receptor-mediated cytokine signaling axis, and maintained EBV antigen-specificity and cytotoxicity. As a result, C7R-EBVSTs showed greater proliferation than NT-EBVSTs over multiple weekly antigen stimulations in the presence and absence of exogenous cytokines. Finally, C7R increased the rate of autologous tumor clearance in our NSG mouse model.

While C7R enhanced EBVST proliferation, it did not produce uncontrolled T-cell outgrowth either in vitro or in our in vivo models in line with the lack of autonomous growth reported by Shum et al., 2017 [33], who showed that prolonged C7R-CAR-T cell expansion was dependent on antigen simulation and co-stimulation. Notably, there was no weight loss in mice receiving C7R-EBVSTs. Since virus-specific T-cells (VSTs) target foreign antigens and have been proved to be safe in multiple clinical trials [5,9,36], the enhancements provided by the C7R should not produce off-target toxicity. The cell-intrinsic cytokine signaling provided by C7R circumvents the cytokine-related adverse toxicity observed with strategies providing cytokine supplementation or transgenic cytokine production by therapeutic T-cells [19,20,28,37] and avoids potential growth promotion of inhibitory cells in the TME or of tumor cells themselves.

IL-7 cytokine-driven STAT5 activity is negatively regulated by the suppression of cytokine signaling-1 (SOCS-1) and/or dephosphorylation by SHP2 [38], therefore one potential concern of expressing constitutively active cytokine signaling receptors is the eventual exhaustion and dysfunction of T-cells. This concern was diminished by our observation that the frequency of TCM and TEM populations were similar in C7R- and NT-EBVSTs on day 16, and because C7R enhanced EBVST persistence after long-term antigen exposure both in vitro and in our murine xenograft model. Further, we observed a higher frequency of T_CM_ in C7R-EBVSTs cultured in the presence or absence of cytokines compared to NT-EBVSTs after long-term in-vitro culture, suggesting that C7R supports and maintains T_CM_ subsets.

STAT5-mediated signaling in T-cells provides resistance to immunosuppressive agents such as M2 macrophages, PD-1-PD-L1 interaction, and TGF-β within the tumor microenvironment [39,40]. Shum et al. reported the downregulation of the TGF-β type 2 receptor by C7R in GD2-CAR T-cells after exposure to GD2-expressing tumor cell lines in vitro [33]. Our preliminary data indicate that C7R protected EBVSTs from the suppressive effects of autologous M2-macrophages, and decreased the frequency of apoptotic cells post 72-h culture in the presence of recombinant TGF-β. These observations suggest that the constitutive STAT5 phosphorylation mediated by C7R may also confer resistance to immunosuppressive elements within the lymphoma microenvironment.

In summary, we have demonstrated the feasibility of expressing C7R in EBVSTs and its ability to enhance the persistence and potency of EBVSTs targeting EBV-associated lymphoma. Though our study is limited by our murine model in assessing the safety profile of C7R-EBSVTs, the lack of autonomous outgrowth of C7R-expressing T-cells in both CAR T- and EBVSTs supports the clinical translation. Nor could our immunodeficient murine model determine the ability of the C7R to counteract a fully functional TME. If successful in improving the clinical potency of EBVSTs for lymphoma, the C7R could be applied to EBVSTs targeting more intractable EBV-associated malignancies, such as nasopharyngeal carcinoma (NPC) and gastric adenocarcinoma (GC), as well as other oncogenic viruses such human papillomavirus (HPV). Future directions will investigate whether C7R provides resistance to specific immunosuppressive components of the TME such as ROS, IDO, NOS, suppressive cytokines (IL-10, IL-13), or inhibitory ligands (e.g., PDL-1). C7R-EBVSTs are currently under clinical and immunological evaluation in patients with EBV+ lymphoma (NCT04664179) [6]. Follow-up studies on patient responses and functional assays to determine T-cell persistence will determine if C7R expression can indeed enhance the persistence of EBSVTs in patients.

## 4. Materials and Methods

### 4.1. Blood Donors and Cell Lines

Blood samples were collected with informed consent from healthy EBV seropositive individuals based on Baylor College of Medicine Institutional Review Board (IRB)-approved protocols. Peripheral blood mononuclear cells (PBMCs) were isolated using Lymphoprep gradients (Axis-Shield, Oslo, Norway) and used for the generation of virus-specific T cells (VSTs) and activated T cells (ATCs). HLA typing was performed by the Houston Methodist HLA Laboratory.

### 4.2. CD3 and CD28-Activated T-Cells (ATCs) for Use as Antigen-Presenting Cells (APCs)

PBMCs were stimulated using CD3 (from the OKT3 hybridoma cell line, ATCC# CRL 8001, Manassas, VA, USA) and CD28 antibodies (Becton Dickinson BD, Franklin Lakes, NJ, USA) in the presence of interleukin-2 (IL-2) (NIH, Bethesda, MD, USA) at 50 units per mL following established procedures as previously documented and consistent with standard laboratory practices [6,34]. Prior to use as antigen-presenting cells (APCs) to stimulate EBVSTs during their second stimulation, activated T cells (ATCs) were re-stimulated with CD3/CD28 antibodies to upregulate costimulatory molecules and then pulsed with a mastermix of EBV T2-Ags pepmixes. They were then irradiated at 30 Gy using an RS2000 X-ray irradiator (RadSource, Suwanee, GA, USA), washed, resuspended in VST medium, and used as APCs.

### 4.3. Pepmixes

Overlapping peptide libraries (15 mer peptides overlapping by 11 amino acids) spanning the complete protein sequences of T2-Ags (EBNA-1, LMP-1, and LMP-2) were purchased from JPT technologies (Berlin, Germany), reconstituted at 200 micrograms/mL in DMSO (Sigma-Aldrich, St. Louis, MO, USA), and stored at −80 °C. Pepmixes were thawed and reconstituted at the appropriate concentration as mentioned below for each experimental use.

### 4.4. Cell Culture Media

ATCs and EBVSTs were cultured in VST medium (RPMI 1640 (HyClone Laboratories Inc., Logan, UT, USA), supplemented with 45% Click’s medium (Irvine Scientific, Santa Ana, CA, USA), 200 mM GlutaMAX TM-I (Gibco Life Technologies, Grand Island, NY, USA), and 10% Fetal Bovine Serum (HyClone Laboratories Inc., Logan, UT, USA). K562cs and LCLs were cultured in RPMI 1640 and enriched with 10% fetal bovine serum and 200 mM Glutamax^TM^.

### 4.5. LCL Generation

EBV-transformed B-lymphoblastoid cell lines (EBV-LCLs) were generated by infecting healthy donor PBMCs with concentrated virus from the B95-8 strain of EBV in the presence of 1 mg per mL of cyclosporin A, followed by culturing allowing the outgrowth of EBV-LCLs, as described previously [41,42].

### 4.6. Costimulatory Cell Lines

The K562 costimulatory cell line (K562cs) was derived from a chronic erythroid leukemia cell line that lacks HLA class I and II molecules by lentiviral transduction with CD80, CD83, CD86, and 4-1BB ligand genes, and was generously provided by Dr. Carl June from the University of Pennsylvania, Perelman School of Medicine. In some experiments, the ULCL was used for co-stimulation in place of K562cs. The ULCL lacks HLA expression and was created within our research center using CRISPR-Cas9 gene-editing techniques.

### 4.7. Generation of Retroviral Vectors

pSFG.ΔCD34-IL7R* (pSFG.C7R): A cDNA encoding a mutant IL-7Rα with a *TTGTCCCAC* insertion between base pairs 731 and 732 (IL7R*) was synthesized (Genscript, Piscataway, NJ, USA) and cloned in an SFG vector backbone. The IL7R* cDNA and the entire extracellular domain of CD34 (ΔCD34) are published in Shum et al. 2017 [33]. Transient retroviral supernatants were produced by co-transfection of NIH 293T cells with the MoMLV gag/pol expression plasmid PeqPam3(-env), the RD114(-env) expression plasmid RDF, and pSFG.C7R at a ratio of 3:2:3, respectively, with a total of 10 μg DNA using GeneJuice reagent (Calbiochem). Supernatants were harvested 48 h after transfection, filtered (using a 0.45 μm filter), snap-frozen, and stored at −80 °C in 5mL aliquots.

### 4.8. Generation of Irradiated, Antigen-Presenting Cell Complex for the Second and Subsequent Stimulations of EBVSTs

Autologous ATCs were pulsed with the EBV pepmix cocktail at 10 ng/1 million cells concentration for 30 min to an hour at 37 °C, then irradiated at 30 Gray. Costimulatory cells (K562cs or ULCLs) were irradiated at 100 Gray. Both cells were then washed, resuspended, and plated with EBVSTs at the ratio mentioned below.

### 4.9. EBVST Generation

PBMCs were stimulated with the EBV T2-Ag (EBNA1, LMP1, and LMP2) pepmix cocktail at 10 ng/million PBMCs, and expanded using IL-15 (100 ng/mL) (PeproTech, Rocky Hill, NJ, USA) and IL-7 (5 ng/mL) (PeproTech) [6,34]. On day 9, expanded cells received a second stimulation (S2) by coculturing EBVSTs with irradiated, pepmix-pulsed, autologous ATCs and irradiated K562cs or ULCLs at an EBVST:ATCs: K562cs/ULCL ratio of 1:1:5 [6,43] in the presence of IL-15 (100 ng/mL) and IL-7 (5 ng/mL). The culture was further expanded through necessary splits and cytokine feeding, and analyzed for phenotype, specificity, and function on day 16 post S1, unless specified otherwise. Despite the likely presence of some bystander cells that do not recognize EBV antigens, we refer to these lines as EBVSTs [6].

### 4.10. Transduction of EBVSTs with C7R

To generate C7R-EBVSTs, pepmix-stimulated PBMCs were transduced with pSFG.C7R retroviral supernatant in the presence of IL-15 (100 ng/mL) and IL-7 (5 ng/mL) four days after the first stimulation (S1). To this end, non-tissue-culture treated 24 well plates were coated with retronectin (Takara Bio, City State, San Jose, CA, USA) according to the manufacturer’s instructions. After washing the coated plates, 1 mL of C7R retroviral vector containing supernatant was transferred per retronectin-coated well, and plated and centrifuged for 90 min at 2000× *g*. After washing, EBVSTs were added at 0.3 × 10^6^ cells per well and centrifuged for 5 min at 400× *g*, before returning to culture.

### 4.11. Immunophenotyping

To analyze phenotypic markers as demonstrated in Figure 1, Figure 2 and Figure 3, T-cells were stained with antibodies to human CD3, CD34, CD4, CD8, CD56, CD45RA, CD45RO, CCR7, and CD62L (BioLegend, San Diego, CA, USA and BD Biosciences, Franklin Lakes, NJ, USA) as previously described [6,33].The stained cells were acquired using the Gallios Flow Cytometer or BD FACS CantoII, and the results were analyzed using Kaluza software version 2.1 (Beckman Coulter, Inc., Brea, CA, USA) or FlowJo analysis software version 7.6.5 and 10.8.1 (FlowJo, LLC, Ashland, OR, USA).

### 4.12. Phosphorylated-STAT5 Assay

Phosphorylated STAT5 expression in NT- and C7R-EBVSTs for Figure 1 was analyzed as previously described [33]. In short, on day 16, NT-EBVSTs and C7R-EBVSTs were harvested, washed, and plated at a concentration of 2 × 10^6^ cells per well in a 24-well tissue culture plate without cytokines. After 72 h, the cells were washed with cold flow buffer (PBS containing 5% FBS), then 100 μL of Fix & Perm Reagent A (Life Technologies) was added followed by gently vortexing, and incubation at room temperature for 3 min. Then, 3 mL of ice-cold methanol was slowly added to the tube while continuously vortexing. The tubes were further incubated at 4 degrees C for 10 min, then centrifuged; the methanol was removed, and the cells were washed again with cold flow buffer. To analyze specific markers, 100 μL of Fix & Perm Reagent B (Life Technologies) and 5 μL of anti-STAT5 antibody (BD Biosciences, Franklin Lakes, NJ, USA) were added to the cells. The cells were gently vortexed and incubated in the dark for 30 min at room temperature. Subsequently, the cells were washed once more with a cold flow buffer and immediately subjected to flow cytometric analysis.

### 4.13. Enzyme-Linked Immunospot (ELISpot) Assay

The frequency of T2-Ags-specific T-cells within the VST population, as demonstrated in Figure 2 and Figure 3, was assessed using IFN-γ ELISpot assays. Anti-human IFN-γ mAb 1-D1K (Mabtech, Cincinnati, OH, USA) was coated in 96-well MultiScreen HTS IP plates (EMD Millipore, Burlington, MA, USA) and incubated overnight at 4 °C. VSTs were seeded at 1 × 10^5^ cells per well in duplicates and stimulated with specified pepmixes at 100 ng/1 × 10^5^ cells or medium alone. Following 16–24 h of incubation at 37 °C in 5% CO_2_, plates were washed and exposed to anti-human IFN-γ mAb 7-B6-1-Biotin (Mabtech, Cincinnati, OH, USA) for 2 to 48 h at 37 °C. Avidin–peroxidase complex (Vector Laboratories, Burlingame, CA, USA) was added for 1 h at room temperature after another wash step. Plates were developed using 3-amino-9-ethylcarbazole (AEC) substrate from Sigma (St. Louis, MO, USA), followed by overnight drying. The quantification was performed externally at Zellnet Consulting (Fort Lee, NJ, USA), or in-house using the Mabtech IRIS–ELISpot reader (Mabtech, Cincinnati, OH, USA). Spot-forming cells (SFCs)/10^5^ were calculated to determine the number of cells releasing IFN-γ in response to viral antigen pepmixes, with negative control values subtracted.

### 4.14. Cytotoxicity Assay

In-vitro cytolytic specificity of EBVSTs, as evaluated in Figure 2, was assessed using a standard 4 h ^51^Cr chromium release assay. Autologous ATCs, either unpulsed or pulsed with pepmixes at a concentration of 10 ng/1 million cells, were labeled with ^51^Cr sodium chromate during a one-hour incubation at 37 °C. After labeling, target cells were washed thrice, resuspended in VST medium, and used as targets for EBVSTs at effector; target (E: T) ratios of 20:1, and 5:1. Medium or 1% Triton X-100 (Sigma-Aldrich, St. Louis, MO, USA) were used to attain spontaneous and maximum release, respectively. Following a 4–6 h co-culture, the supernatant was collected, and ^51^Cr released was analyzed using a gamma counter. The percent specific lysis was computed from the mean of triplicates using the formula [(experimental release − spontaneous release)/(maximum release − spontaneous release)] × 100.

### 4.15. In-Vivo Murine Model

To evaluate the anti-tumor efficacy, and persistence of EBVSTs in-vivo, as described in Figure 4, female 4 to 6 weeks NSG mice (NOD.Cg-Prkdcscid Il2rgtm1Wjl/SzJ), (Jackson Laboratory, Bar Harbor, ME, USA) were subcutaneously engrafted with EBV-LCLs suspended within 200 µL Matrigel matrix (Corning, Tewksbury, MA, USA). Ten days later, when tumors were palpable, C7R- or NT-EBVSTs expressing GFP-FF-Luc from a retroviral vector were introduced intravenously (i.v.). Tumor volume was measured using an external caliper and computed using the equation: tumor volume = ½ (length) * (width)^2^. Bioluminescence measurements were taken with an IVIS Imaging system (Caliper Life Sciences, Hopkinton, MA, USA) following a 10 min wait period after the intraperitoneal administration of 150 mg/kg D-luciferin (Xenogen) per mouse. Total luminescence over the tumor area was visualized and quantified using Living Image software version 4.7.3 (PerkinElmer, Waltham, MA, USA), with the region of interest (ROIs) encompassing the tumor region. All procedures were conducted in accordance with the Institutional Animal Care and Usage Committee (IACUC) approved protocol #AN5551 at Baylor College of Medicine.

### 4.16. Statistical Analysis

We used GraphPad Prism 7 (GraphPad Software, Inc., La Jolla, CA, USA) for statistical analysis, using paired Students *t*-test and/or as indicated in figure legends. Data are plotted as mean ± SEM unless otherwise indicated. Significance is denoted by *p* < 0.05 (*), *p* < 0.01 (**), *p* < 0.001 (***) unless otherwise indicated.

## 5. Patents

CMR, BO, and TS are co-authors of patent application WO2018038945A1, and the C7R technology is licensed to Gracell Biotechnologies (Suzhou, China).

## Data Availability

All data are available in the main text o Any additional data of interest can be available upon request.
